# Papillary Thyroid Carcinoma, Bilateral Macronodular Adrenal Cortical Disease-Related Cortisol Excess, and Femoral Enchondroma: A Novel Phenotype–Genotype Based on Next-Generation Sequencing (Variants of *APC, MSH6*, and *CACNA1S* Genes)

**DOI:** 10.3390/diagnostics16081185

**Published:** 2026-04-16

**Authors:** Mara Carsote, Sorina Violeta Schipor, Anda Dumitrascu, Ana-Maria Gheorghe, Oana-Claudia Sima, Dana Manda, Mihai Costachescu, Andrei Muresan, Emi Marinela Preda, Dana Terzea

**Affiliations:** 1 Department of Endocrinology, “Carol Davila” University of Medicine and Pharmacy, 020021 Bucharest, Romania; carsote_m@hotmail.com; 2 Department of Clinical Endocrinology V, “C.I. Parhon” National Institute of Endocrinology, 011863 Bucharest, Romania; ana-maria.gheorghe@drd.umfcd.ro (A.-M.G.); oana-claudia.sima@drd.umfcd.ro (O.-C.S.); 3 Department of Research, “C.I. Parhon” National Institute of Endocrinology, 011863 Bucharest, Romania; dana.manda@parhon.ro (D.M.); andrei.muresan@parhon.ro (A.M.); 4 Department of Radiology and Medical Imaging, “C.I. Parhon” National Institute of Endocrinology, 011863 Bucharest, Romania; anda.dumitrascu@gmail.com; 5 PhD School of “Carol Davila” University of Medicine and Pharmacy, 020021 Bucharest, Romania; 6 Department of Radiology and Medical Imaging, “Dr. Carol Davila” Central Military University Emergency Hospital, 010825 Bucharest, Romania; 7 Department of Radiology, “Carol Davila” University of Medicine and Pharmacy, 050474 Bucharest, Romania; emi.preda@umfcd.ro; 8 Department of Radiology and Medical Imaging, “Foisor” Clinical Hospital of Orthopaedics, Traumatology and Osteoarticular TB, 021382 Bucharest, Romania; 9 Department of Pathology, “C.I. Parhon” National Institute of Endocrinology, 011863 Bucharest, Romania; danaterzea@gmail.com; 10 Oncoteam Diagnostics, 012244 Bucharest, Romania

**Keywords:** next-generation sequencing, thyroid cancer, adrenal tumor, ACTH, cortisol, thyroglobulin, adrenalectomy, CT, MRI, NGS, PTC

## Abstract

This case highlights a novel genotype–phenotype correlation in the field of endocrinology. Specific endocrine and imaging assessment, in addition to next-generation sequencing (NGS), was performed on the Illumina MiSeq platform, using a TruSight One Sequencing Panel kit for genomic analysis of coding regions of 4813 genes. A 54-year-old female was confirmed with a papillary thyroid carcinoma after total thyroidectomy and underwent radioiodine ablative therapy. Three years later, a left femoral enchondroma of almost 3 cm was identified at computed tomography (CT) scan and magnetic resonance imaging (MRI). She experienced hypertension (in addition to obesity, dyslipidaemia and impaired glucose tolerance) and was later confirmed with ACTH-independent cortisol excess [lack of cortisol suppression at 1 mg dexamethasone testing of 13.9 (normal < 1.8 µg/dL)], noting bilateral adrenal tumors, of 4.7 cm (right), respectively, and of 1.6 cm (left) at CT. Right laparoscopic adrenalectomy was performed with post-operative adrenal insufficiency, requiring glucocorticoid replacement and stopping the anti-hypertensive medication. Pathology report confirmed an adrenocortical adenoma (a Ki67 proliferation index of 2%). Noting the unusual association of the mentioned conditions, NGS was performed in the peripheral blood and identified a heterozygote missense variant of the *APC* gene (c.5759G>A, p.Arg1920Gln), a heterozygote missense variant of the *MSH6* gene (c.2092C>G, p.Gln698Glu), and an incidental additional finding: a heterozygote stop gain pathogenic variant of the *CACNA1S* gene (c.2707C>T, p.Arg903*). The first two are currently classified as variants of uncertain significance. Whether the co-presence of a triple mutation may change the clinical picture and the life-long outcomes across reciprocal influence is still an open matter. Further research will point out the clinical implications of this genotype–phenotype association, which, to our best knowledge, has not been previously reported.

**Figure 1:**
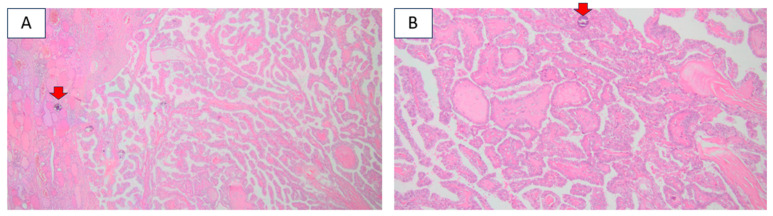
This is a 54-year-old female who was accidentally identified with a thyroid nodule of 3.6 cm in diameter at anterior neck ultrasound. She underwent total thyroidectomy with confirmation of a differentiated thyroid carcinoma. [Histological report (hematoxylin–eosin) confirmed a papillary, sclerosant, multifocal carcinoma with several foci under 1 cm (centimeter), the largest of 0.7 by 0.6 cm, respectively, of 0.6 by 0.6 cm, with psammoma bodies (red arrows). (**A**) Magnification 4×; (**B**) magnification 10×.] Radioiodine therapy (100 mCI) was offered to the patient in addition to life-long Thyroid-Stimulating Hormone (TSH)-suppressive levothyroxine therapy, which achieved undetectable serum thyroglobulin levels within the following year.

**Figure 2:**
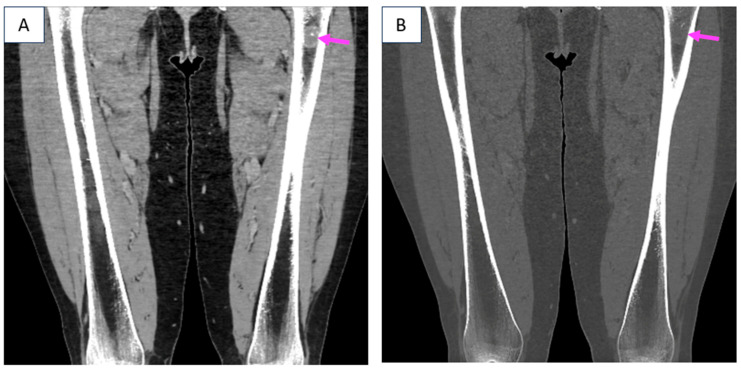

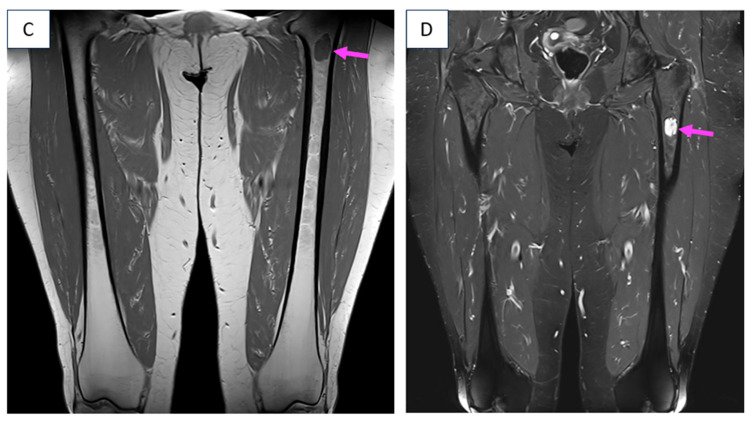
Later on, at the age of 57, the patient experienced non-specific, mild, persistent bone and joint pain and further investigations lead to the identification, not only of incipient arthrosis, but, also, of a left femoral tumor with cartilaginous structure and benign appearance (an enchondroma) at computed tomography (CT) and magnetic resonance imaging (MRI) [(**A**,**B**) CT scan (native phase): bone tumor in the diaphysis of the left femur of 1.9 by 2.5 cm, without cortical involvement (pink arrow); (**A**) coronal plane; (**B**) coronal plane with bone window. (**C**,**D**) Contrast-enhanced MRI scan: bone tumor in the diaphysis of the left femur of 1.9 by 1.8 by 2.9 cm with polycyclic margins, with T1 hypo-signal and T2 hypersignal, without cortical thinning, suggestive of enchondroma (pink arrow); (**C**) T1 WI HSE, coronal plane; (**D**) T2 FSE, coronal plane].

**Figure 3:**
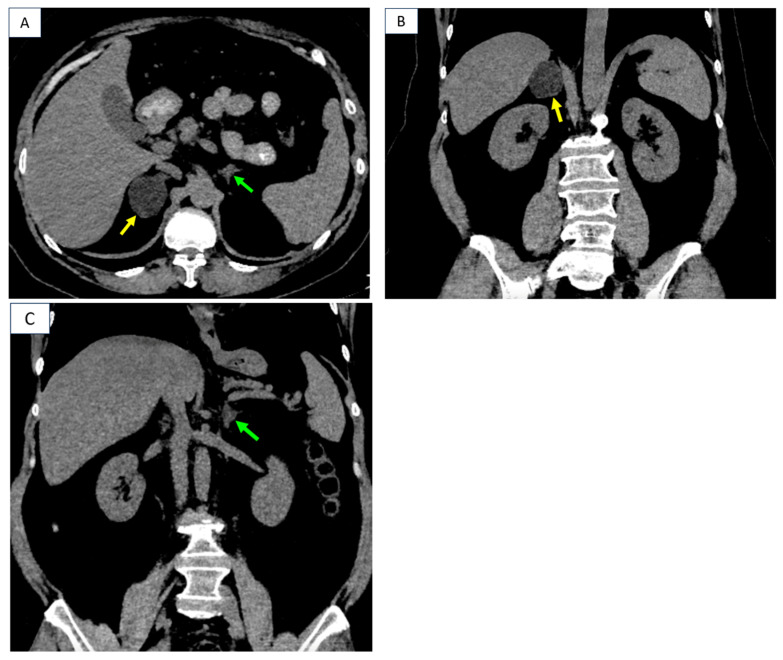
In the meantime, since the age of 56, she was confirmed with arterial hypertension, requiring re-assessment at a tertiary center of endocrinology. The clinical aspects, while not being highly suggestive for a typical Cushing’s syndrome, pointed out metabolic complications in terms of obesity (a body mass index of 36 kg/sqm); dyslipidemia [increased triglycerides of 205 (normal < 200) mg/dL, and total cholesterol of 248 (normal < 200) mg/dL]; impaired glucose tolerance based on a 2 h glycemia during 75 g oral glucose tolerance test of 147 mg/dL, mildly elevated glycated hemoglobin A1c of 6.4 (normal: 4.8–5.9) %, and insulin resistance according to a HOMA-IR (Homeostasis Model Assessment Model of Insulin Resistance) level of 5.3 (insulin resistance at HOMA-IR above the cutoff 2). Hormonal panel was conclusive for Adrenocorticotropic Hormone (ACTH)-independent (autonomous) cortisol excess based on: suppressed baseline morning ACTH of 2.7 (normal: 7.2–63.3) pg/mL, normal morning plasma cortisol of 13.1 (normal: 4.82–19.5) µg/dL without suppression at 1 mg overnight dexamethasone testing according to the second-day plasma cortisol level of 13.9 (normal < 1.8) µg/dL. The central Dual-Energy X-Ray Absorptiometry (DXA) scan excluded osteoporosis/osteopenia (lowest T-score of +0.8 SD, and bone mineral density of 1.098 g/sqcm at total hip), and the trabecular bone score of 1.388 was normal. CT showed bilateral adrenal tumors [3. CT scan (native phase): right adrenal tumor (yellow arrow) of 3.7 by 4.4 by 4.7 cm with negative density, heterogeneous, in close contact with the inferior vena cava, the upper pole of the right kidney, the fifth hepatic segment and the right diaphragmatic crus and left adrenal tumor of 1.1 by 1.6 cm (green arrow); (**A**) axial plane; (**B**) coronal plane; (**C**) coronal plane].

**Figure 4:**
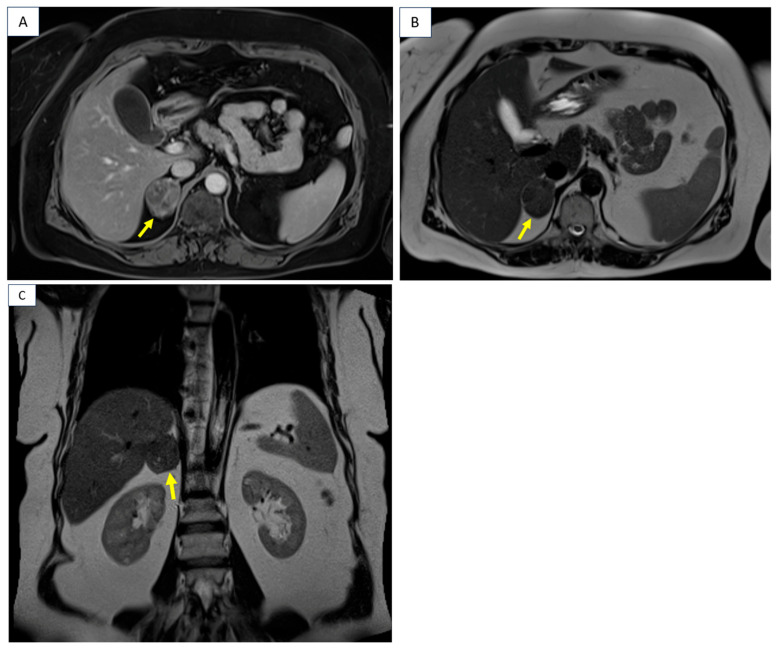
MRI confirmed similar features of the adrenal tumors. [4. Contrast-enhanced MRI scan: right adrenal tumor of 4.2 by 3.3 by 4.5 cm with intracellular lipid content and no restricted diffusion on diffusion-weighted imaging (DWI) (yellow arrow); (**A**) T1 VIBE Dixon, axial plane; (**B**) T2 HASTE, axial plane; (**C**) T2 HASTE, coronal plane].

**Figure 5:**
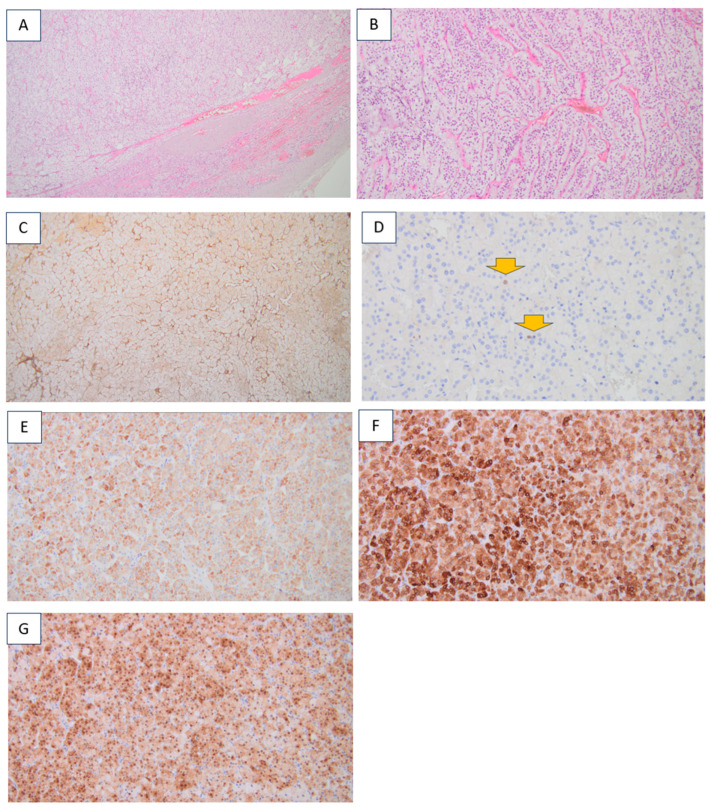
Due to the cortisol over-production with bilateral adrenal gland involvement, but with one tumor significantly larger than the other (>3 cm difference), and the fact that the largest diameter was above the cutoff of 4 cm in the right tumor mass, unilateral laparoscopic adrenalectomy was recommended and performed with a good outcome. Post-operative adrenal insufficiency developed, requiring glucocorticoid replacement for a few months in addition to an improvement in the high blood pressure, so that the anti-hypertensive drugs were no longer necessary. Pathology report confirmed an adrenocortical adenoma [(**A**–**C**). Histological analysis: (**A**) hematoxylin–eosin (magnification 4×); (**B**) hematoxylin–eosin (magnification 10×); (**C**) intact reticulin network (magnification 4×); (**D**–**G**) Immunohistochemistry analysis (Weiss score of 0); (**D**) Ki67 proliferation index of 2% (orange arrows) (magnification 20×); (**E**) medium-level staining of melan A in the cytoplasm of tumor cells (magnification 10×); (**F**) strong cytoplasmic staining of inhibin in tumor cells (magnification 10×); (**G**) medium to high cytoplasmic and strong nuclear staining of calretinin in tumor cells (magnification 10×)].

**Figure 6:**
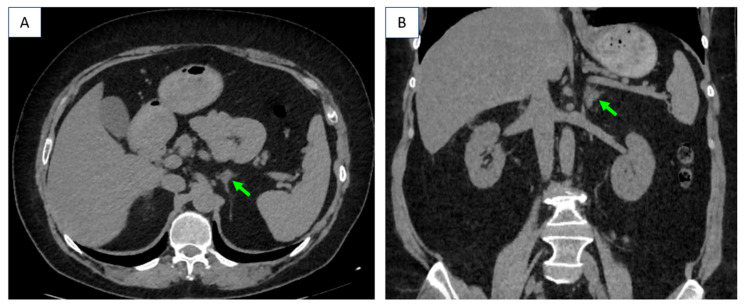
Six months after unilateral adrenalectomy, CT showed stationary left adrenal tumor (of 1.2 by 1.5 cm) [CT scan native phase highlights the tumor (green arrow): (**A**) axial plane; (**B**) coronal plane].

**Figure 7:**
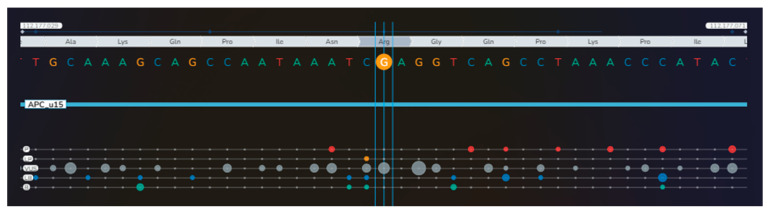
Noting the unusual association of the mentioned conditions, next-generation sequencing (NGS) was performed in the peripheral blood on the Illumina MiSeq platform, using TruSight TruSeq One Sequencing Panel kit (Illumina, San Diego, CA, USA) for genomic analysis of coding regions of 4813 genes. Alignment and Variant Calling were performed using BWA and GATK in Local Run Manager (Illumina). Variants were classified using the American College of Medical Genetics and Genomics (ACMG) criteria and databases, including ClinVar, HGMD, and gnomAD. For tertiary analysis, we used Geneyx Analysis software v6.2 (Geneyx Genomex Ltd., Wilmington, DE, USA), which guided prioritization of genes related to this phenotype (papillary thyroid carcinoma, bilateral macronodular adrenal cortical disease and femoral enchondroma). We identified a heterozygote missense variant of the *APC* gene (c.5759G>A, p.Arg1920Gln) [P = pathogenic (red bullets); LP = likely pathogenic (orange bullets); VUS = variant of uncertain significance (gray bullets); LB = likely benign (blue bullets); B = benign (green bullets); the dimension of the bullets depends on the number of submissions with the same classification in ClinVar for that specific variant] [1] (Table S1).

**Figure 8:**
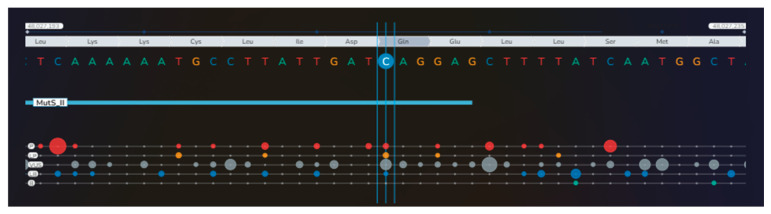
Moreover, a heterozygote missense variant of the *MSH6* gene (c.2092C>G, p.Gln698Glu) was also identified: P = pathogenic (red bullets); LP = likely pathogenic (orange bullets); VUS = variant of uncertain significance (gray bullets); LB = likely benign (blue bullets); B = benign (green bullets); the dimension of the bullets depends on the number of submissions with the same classification in ClinVar for that specific variant] [1] (Table S2).

**Figure 9:**
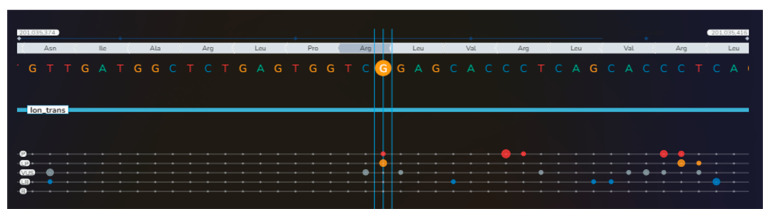
Also, an incidental additional finding included a heterozygote stop gain pathogenic variant of the *CACNA1S* gene (c.2707C>T, p.Arg903*) [P = pathogenic (red bullets); LP = likely pathogenic (orange bullets); VUS = variant of uncertain significance (gray bullets); LB = likely benign (blue bullets); B = benign (green bullets); the dimension of the bullets depends on the number of submissions with the same classification in ClinVar for that specific variant] [1]. Particular aspects of this case involve the unusual association of endocrine and non-endocrine tumors/malignancy that, to our best knowledge, has not been reported prior, especially in relation to a novel mutational landscape across NGS. Although there are no cases with all three tumors in one individual, the association of any two tumors has been identified across distinct genetic backgrounds from the current one. For instance, the association between enchondromas and adrenal tumors was found in Maffucci syndrome. The underlying genetic defects are still an open matter (probably involving *IDH1* and *IDH2* genes) [2,3,4,5]. Moreover, enchondromas, especially pulmonary enchondromas, and adrenal tumors are part of the Carney triad [6]. Soft tissue tumors and bone tumors (e.g., osteomas) might be associated with familial adenomatous polyposis underlying *APC* pathogenic variants [7,8], noting that, traditionally, the tumor-suppressor gene (located on chromosome 5) is linked with this hereditary (autosomal dominant) syndrome [9]. In this case, the gastroenterological evaluation showed no pathological findings, nor was the patient’s family known to have a specific hereditary syndrome. Notably, a recent report of a synchronous thyroid malignancy was connected to a novel *APC* pathogenic variant (c.2929delG frameshift deletion) [10], while non-medullary (particularly, cribriform-morular variant of the papillary type) thyroid cancer was found to be associated with the syndrome in exceptional population subgroups [11,12,13]. Ongoing genetic research is mandatory to further pinpoint the role of the *APC* gene in the development of differentiated thyroid cancer. However, in the present case, the p.R1920Q variant of *APC* gene is currently classified as variant of uncertain significance (VUS) based on extremely low frequency in gnomAD population databases (gnomAD maximal non-founder subpopulations frequency of 0.003%), so it cannot be considered a common polymorphism and the causal relationship with the phenotype on point has not been established so far. Bioinformatic prediction tools show conflicting evidence for functional effects of this missense variant (REVEL: benign, SIFT: benign, Mutation Taster: disease-causing, Polyphen2: deleterious, GenoCanyon: deleterious), and aggregated in silico prediction suggests an uncertain effect. This variant has not been reported in the literature in individuals affected by *APC*-related conditions, but it is cited in ClinVar by five reputable databases/clinical laboratories and reported as VUS [14]. Interestingly, malignant bone tumors have been rarely associated with *MSH6* pathogenic variants. Yet, in this adult female, the imaging assessment was suggestive of a benign tumor (further investigations were declined by the patient) [15]. Pathogenic variants of the *MSH6* gene, a DNA mismatch repair gene, are described in Lynch syndrome, an autosomal dominant hereditary cancer syndrome, typically involving colorectal and endometrial carcinoma, but a variable prevalence of gastric, hepatobiliary, pancreatic, and ovarian malignancies has been co-reported, too [16,17,18]. Moreover, some *MSH6* variants have been described in a subgroup of individuals showing both intra-adrenal and extra-adrenal paraganglioma [19], while the oncocytic variant of the adrenocortical carcinoma was diagnosed in one patient with Lynch syndrome [20]. *MSH6* gene might also be involved in thyroid pathology, as suggested by a retrospective analysis which identified *MSH6* variants in 36% of patients with Lynch syndrome in addition to thyroid nodules, underlying adenomas and carcinomas [21]. In this instance, the p.Q1920E variant of the *MSH6* gene is also classified as VUS based on extremely low frequency in gnomAD population databases (0.002%) and in silico predictors with an aggregated score suggesting an uncertain effect on protein. This variant has twelve submissions in ClinVar, eleven of them with VUS annotation, and it has been reported in several populations diagnosed with Lynch syndrome: French [22], Danish [23], and Cypriot [24], noting that currently, it remains classified as VUS [25]. Anomalies of the calcium receptor may be rarely involved in the adrenal tumorigenesis, as shown by studies that linked pathogenic variants of genes encoding the T-type calcium channel, such as *CACNA1H*, to primary hyperaldosteronism and paraganglioma [26,27]. On the other hand, the *CACNA1S* gene encodes the Cav1.1 L-type calcium channel, having the highest expression in muscle cells, and being typically involved in hypokalaemic periodic paralysis, malignant hyperthermia and congenital myopathy [28,29,30,31]. In addition, some authors identified variants in the *CACNA1S* gene in patients with endocrine ailments. For instance, a unilateral adrenal nodule was confirmed in a 43-year-old male patient with hypokalaemic periodic paralysis, harboring a missense variant of the *CACNA1S* gene, as similarly found in the other five adults (family members) [32]. A connection between thyrotoxic periodic paralysis and *CACNA1S* polymorphisms was hypothesized to increase the calcium channel susceptibility to the thyroid hormone excess [33]. Previous data also linked the *CACNA1S* gene to hyperthyroidism in children [34]. Following ACMG recommendations [35], we tested a panel of 84 genes for incidental findings, and we identified a pathogenic variant in the *CACNA1S* gene (c.2707C>T, p.Arg903*), a variant which was previously linked to malignant hyperthermia susceptibility, hypokalemic periodic paralysis or congenital myopathy [36,37]. None of these conditions were presented in this patient, and the exact clinical consequence of this *CACNA1S* variant remains an open issue, especially in relation to the co-presence of the other two VUS of the *APC* and *MSH6* genes. On a final note, the wide clinical spectrum (thyroid–adrenal–bone) seems to be a consequence of these genetic disturbances, which, currently, are more or less understood. Yet, globally, papillary thyroid cancer remains the most common endocrine malignancy and an incidental diagnosis cannot be entirely ruled out at this point. Moreover, 1% to 5% of the adults aged 50 or older might suffer from (ACTH-independent) autonomous cortisol secretion due to adrenal tumors, a prevalence which is even higher in the population subgroup showing metabolic complications (e.g., dyslipidemia, obesity, diabetes, and hypertension). The genetic background represents a cutting-edge exploration nowadays, especially in bilateral macronodular adrenal cortical disease [38]. On a practical level, long-term TSH suppressive therapy might pose additional cardiovascular risks across the life span, while the contra-lateral adrenal tumor may prove secretory, as well. Whether the co-presence of a triple mutation potentially changes the clinical picture and the life-long outcomes across reciprocal influence is still an open matter. Further research will point out the clinical implications of this novel genotype–phenotype association and potential costs involving NGS testing in such patients with further protocols implementation amid daily endocrine practice. Overall, this vignette is meant to raise awareness among practitioners with multidisciplinary backgrounds in the field of endocrine pathology and genetic connections.

## Data Availability

The original contributions presented in this study are included in the article/Supplementary Material. Further inquiries can be directed to the corresponding author.

## Supplementary Materials

The following supporting information can be downloaded at: https://www.mdpi.com/article/10.3390/diagnostics16081185/s1, Table S1: In-Silico Analysis Scores for APC gene: c.5759G>A, chr5-112177050 G>A, p.Arg1920Gln, rs587780599. Table S2: In-Silico Analysis Scores for MSH6 gene: c.2092C>G, chr2-48027214 C>G, p.Gln698Glu, rs63750832.

## Abbreviations

The following abbreviations are used in this manuscript:

ACTH	Adrenocorticotropic Hormone
ACMG	American College of Medical Genetics and Genomics
cm	Centimeter
CT	Computed Tomography
DWI	Diffusion-Weighted Imaging
HOMA-IR	Homeostasis Model Assessment Model of Insulin Resistance
MRI	Magnetic Resonance Imaging
NGS	Next-Generation Sequencing
TSH	Thyroid-Stimulating Hormone
VUS	Variant of Uncertain Significance
