# Supplementary material for: Papillary Thyroid Carcinoma, Bilateral Macronodular Adrenal Cortical Disease-Related Cortisol Excess, and Femoral Enchondroma: A Novel Phenotype–Genotype Based on Next-Generation Sequencing (Variants of APC, MSH6, and CACNA1S Genes)
Source: Diagnostics (Basel). 2026 Apr 16;16(8):1185. doi: 10.3390/diagnostics16081185 (PMC13114812; doi:10.3390/diagnostics16081185)
Supplement: Supplementary file 1 [file diagnostics-16-01185-s001.zip › diagnostics-4219866-supplementary.pdf]

Supplemental tables involving the genomic analysis based on next-generation sequencing, which involve the two variants that have been identified and currently they are classified as variants of uncertain significance

Table S1: In-Silico Analysis Scores for *APC* gene: c.5759G>A, chr5-112177050 G>A p.Arg1920Gln, rs587780599.

Method or software	Score
Aggregated prediction	***Uncertain (0.34)***
Functional coding	
REVEL	Benign (Supporting) (0.22)
AlphaMissense	Benign (Moderate) (0.1)
Varity	Benign (low) (0.11)
MutationAssessor	Med (1.96)
SIFT	Benign (Supporting) (0.189)
POLYPHEN2	Deleterious (Supporting) (0.99)
Mutation Taster	Deleterious (1)
FATHMM	Uncertain (-2.55)
DANN	Deleterious (1)
MetaLR	Deleterious (low) (0.51)
PrimateAI	Benign (Supporting) (0.38)
BayesDel	Benign (Supporting) (-0.18)
Splice Altering	
Splice AI:	Benign (0)
Conservation	
GERP	Uncertain (5.14)
Functional Whole Genome	
GenoCanyon:	Deleterious (0.99)
fitCons:	Deleterious (0.65)

Table S2: In-Silico Analysis Scores for *MSH6* gene: c.2092C>G, chr2-48027214 C>G, p.Gln698Glu, rs63750832.

Method or software	Score
Aggregated prediction	***Uncertain (0.55)***
Functional coding	
REVEL	Uncertain (0.46)
AlphaMissense	Benign (Moderate) (0.083)
Varity	deleterious (low) (0.5)
MutationAssessor	Lo (1.5)
SIFT	Benign (0.119)
POLYPHEN2	Uncertain (0.68)
Mutation Taster	deleterious (1.00)
FATHMM	Uncertain (-2.26)
DANN	Deleterious (0.95)
MetaLR	benign (low) (0.39)
PrimateAI	uncertain (0.52)
BayesDel	uncertain (0.05)

Splice Altering	
Splice AI:	Benign (0.04)
Conservation	
GERP	Uncertain (4.05)
Functional Whole Genome	
GenoCanyon:	Deleterious (1)
fitCons:	Deleterious (0.67)
